# Rotating and Neurochemical Activity of Rats Lesioned with Quinolinic Acid and Transplanted with Bone Marrow Mononuclear Cells

**DOI:** 10.3390/bs8100087

**Published:** 2018-09-20

**Authors:** Teresa Serrano Sánchez, María Elena González Fraguela, Lisette Blanco Lezcano, Esteban Alberti Amador, Beatriz Caballero Fernández, María de los Ángeles Robinson Agramonte, Lourdes Lorigados Pedre, Jorge A Bergado Rosado

**Affiliations:** 1 Immunochemical Department, International Center for Neurological Restoration, 25th Ave, Playa, 15805, Havana PC 11300, Cuba; marie@neuro.ciren.cu (M.E.G.F.); robin@neuro.ciren.cu (M.d.l.Á.R.A.); lourdesl@neuro.ciren.cu (L.L.P.); 2 Experimental Neurophysiology Department, International Center of Neurological Restoration (CIREN) Ave. 25 No. 15805 e/158 and 160, Playa, Havana 11300, Cuba; lblanco@neuro.ciren.cu (L.B.L.); jorgebergado@yahoo.com (J.A.B.R.); 3 Molecular biology Department, International Center of Neurological Restoration (CIREN) Ave. 25 No. 15805 e/158 and 160, Playa, Havana 11300, Cuba; alberti@neuro.ciren.cu; 4 Policlínico 26 de Julio, Calle 72 #1313. A. Almendares, Playa, Havana 11300, Cuba; bettycfdez@infomed.sld.cu

**Keywords:** striatum, quinolinic acid, transplantation, mononuclear cell, bone marrow cell, Huntington disease model

## Abstract

Huntington’s disease (HD) is an inherited, neurodegenerative disorder that results from the degeneration of striatal neurons, mainly GABAergic neurons. The study of neurochemical activity has provided reliable markers to explain motor disorders. To treat neurodegenerative diseases, stem cell transplants with bone marrow (BM) have been performed for several decades. In this work we determine the effect of mononuclear bone marrow cell (mBMC) transplantation on the rotational behavior and neurochemical activity in a model of Huntington’s disease in rats. Four experimental groups were organized: Group I: Control animals (*n* = 5); Group II: Lesion with quinolinic acid (QA) in the striatum (*n* = 5); Group III: Lesion with QA and transplant with mBMC (*n* = 5); Group IV: Lesion with QA and transplant with culture medium (Dulbecco’s modified Eagle’s medium (DMEM) injection) (*n* = 5). The rotational activity induced by D-amphetamine was evaluated and the concentration of the neurotransmitter amino acids (glutamate and GABA) was studied. The striatal cell transplantation decreases the rotations induced by D-amphetamine (*p* < 0.04, Wilcoxon matched pairs test) and improves the changes produced in the levels of neurotransmitters studied. This work suggests that the loss of GABAergic neurons in the brain of rats lesioned with AQ produces behavioral and neurochemical alterations that can be reversed with the use of bone marrow mononuclear cell transplants.

## 1. Introduction

Huntington’s disease (HD) has been associated with a degeneration of striatal cells, mainly death of medium spiny GABAergic projection neurons within the caudate nucleus and putamen. Although the most pronounced pathology is observed in the basal ganglia, cell death also occurs early in the cerebral cortex [1]. The experimental models play essential roles in the understanding of disease mechanisms, progression, and drug efficacy testing. The injection of quinolinic acid into the striatum is used as a theoretical model of HD [2]. Quinolinic acid (QA) produces loss of GABAergic neurons; in this way there is an imbalance between the healthy and damaged striatum in response to the action of an intact dopaminergic system, which produces an asymmetric motor response to the action of a dopaminergic agonist, D-amphetamine.

The study of the neurochemical activity in the affected brain tissue is a reliable marker to explain the motor disorders that appear in this disease [3]. No therapy has been shown to delay disease onset or slow progression in humans. Unlike other neurodegenerative entities, pharmacological treatment is only a palliative procedure. Thus, there is an urgent need to identify and validate other treatments. Cells isolated from bone marrow are successfully used for transplantation in experimental models of cranial encephalic trauma [4,5] as well as in striatal ischemia to reduce the motor deficit that appears after damage [6]. Recently, autologous bone marrow stem cell transplantation has been used to reverse the cognitive deficit observed in an experimental model of Huntington’s disease, which demonstrates that bone marrow cell transplantation is able to reduce the cognitive damage that appears in this model [7]. In general, the repair mechanism consists of the potential to develop into many different types of cells (thus serving as a repair system for the body) and the potential to release trophic factors that repair and regulate other compensatory mechanisms in the damaged area. For this reason, in the last decades, neurorestorative treatment has been tried in experimental models that use stem cells from different sources, such as mononuclear bone marrow cells (mBMC) [8,9]. 

In the present study, the objective was to determine the effect of mBMC transplantation on the rotational activity induced by systemic administration of D-amphetamine (5 mg/kg intraperitoneal) and the neurochemical activity in two brain areas: the cerebral cortex and striatum. We show evidence that indicates the transplant may be a viable therapeutic option for Huntington’s disease.

## 2. Materials and Methods

### 2.1. Animals

Adult male Sprague Dawley (SD) rats (total *N* = 20) obtained from the National Center for Laboratory Animals Production (CENPALAB) and weighing 200–250 g (between 9 and 12 weeks of age) were used in this study. Animals were housed in translucent makrolon cages (five animals per cage) under a 12 h light: 12 h darkness cycle with ad libitum access to water and food. 

For the behavior and neurotransmission study, four experimental groups were included (*n* = 5 each): Control (C), Group I; QA lesion (QAL), Group II; QA lesion + mononuclear bone marrow cell transplantation (QA+mBMC), Group III; and QA lesion + Dulbecco’s modified Eagle’s medium (QA+DMEM) injection, Group VI.

### 2.2. Striatal QA Lesions

Unilateral lesions of the right striatum were produced by the intrastriatal injection of QA. Rats were anesthetized with an intraperitoneal (ip) injection of ketamine (50 mg/mL, ip, IMEFA, Havana, Cuba). QA injections were administered with the help of a stereotaxic apparatus (model 900; David Kopf Instruments, Tujunga, CA, USA). Each rat was injected in the striatum with 1.2 μL of QA (125.5 nmol) (Sigma, Saint Louis, MA, USA) using a 30 G Hamilton syringe at the following coordinates: 1.2 mm anterior and 2.8 mm lateral to the bregma, and 5.5 mm below the dura. The toxin was injected over a period of 1 min, and the cannula was left in place for an additional 10 min before being slowly removed.

### 2.3. Obtaining Rat Mononuclear Bone Marrow Cells

The mBMC were isolated from the rat femur as described in the work of Woodbury and colleagues [10]. Male SD rats aged between 32 and 48 days old were anesthetized with an intraperitoneal injection of ketamine (50 mg/mL, ip, IMEFA, Havana, Cuba) and a cut on the skin of the hind limbs was performed, separating the tissue parallel to the bone to extract both femurs; then the animals were euthanized with a lethal overdose of chloral hydrate. The extracted bones were placed for 30 min on a Petri dish containing 0.9% physiologic saline, after which the bone marrow was obtained by flushing with sterile phosphate-buffered saline (PBS; NaCl, 8 g/L; KCl, 0.2 g/L; Na_2_HPO_4_, 1.09 g/L; KH_2_PO_4_, 0.26 g/L, pH 7.2) through one of the femoral epiphyses. The bone marrow was collected in sterile containers to be later washed by centrifugation.

### 2.4. Isolation of Mononuclear Bone Marrow Cells 

The suspension of bone marrow cells was washed three times with 1× PBS by centrifugation for 10 min at 2000 rpm at 20 °C. An aliquot of 2.5 mL of Ficoll–Hypaque was placed on the bottom of a graduated glass tube, on top of which 5 mL of the cellular suspension in PBS was layered. This was centrifuged for 45 min at 2800 rpm at 20 °C.

The mononuclear cell band was extracted with a pipette and washed immediately, discarding the supernatant into a container with hypochlorite and collecting the cellular pellet, which was suspended in 1× PBS and cell viability was determined by trypam blue exclusion.

### 2.5. Transplantation

Four weeks after the QA lesion, the animals to be transplanted with mBMCs were deeply anesthetized as previously described. Rats were placed in the stereotactic apparatus and the skin over the skull was reopened. Using a Hamilton syringe, the mBMC suspension (50,000 cells/μL in DMEM) was injected into the lesioned striatum (in two deposits; 1 μL per deposit) at coordinates slightly different from the ones used for the QA lesions: 0.7 mm anterior from the bregma, 2.8 mm lateral from the midline, and 5.5 and 4.6 mm under the dura surface. Cells were injected slowly over a period of 1 min. The needle was left in place for an additional 10 min following injection and then carefully removed. Sham-transplanted animals (DMEM) received an equal volume of tissue culture medium injected in the same way, at the same stereotactic coordinates. 

### 2.6. Behavioral Tests (Rotating Activity Induced by D-Amphetamine)

The rotational activity induced by D-amphetamine (5 mg/kg, ip, Sigma, St. Luis, MO, USA) [11] was studied one week after the QA injury and one month after the mBMC/ DMEM transplant, using an LE 3806 Electronic Multicounter coupled to LE 902 sensors (PanLAB, Barcelona, Spain) that measured the sense of rotation. The measurements were performed for a period of 90 min.

### 2.7. Obtaining Samples for Neurotransmission Study 

After the behavioral test was over, the rats received an overdose of ketamine (100 mg/mL, ip, IMEFA, Havana, Cuba) and were decapitated. Their brains were extracted and washed with cold 0.9% NaCl, after which the striatum (St) and prefrontal cortex was dissected. The obtained tissue was frozen in liquid nitrogen, weighed and stored at −80 °C for further analysis.

### 2.8. Neurotransmitter Study

The amino acid concentrations in the tissue were determined by high-performance liquid chromatography (HPLC), coupled to a fluorescence detector and using derivatization with o-phthaldialdehyde (OPA). Then, 10 μL of sample was mixed with 10 μL of the OPA derivatizing agent (10 mM OPA dissolved in 0.1 mol/L sodium tetraborate buffer containing 77 mM of 3-mercaptopropionic acid and 10% methanol pH 9.3). The mixture was vortexed for 15 s and the reaction was stopped with 5% acetic acid at 45 s. From this mixture, 20 μL was injected to the chromatograph with a Hamilton syringe. The derivatized amino acids were passed through a reverse phase column (HR-80, 8 cm long with a4.6-mm internal diameter, ESA), with a similar stationary phase precolumn, by means of an isocratic chromatographic pump (Philips PU, Amsterdam, The Netherlands) 4100) and were detected by a fluorescence detector with excitation λ = 340 nm and emission λ = 460 nm (Philips PU 4027). The chromatograms were recorded using the program CHROMATEPC version 4.24 (Philips). A mobile phase composed of 0.1 mol/L NaH_2_PO_4_ and 20% methanol was used to separate the amino acids.

### 2.9. Ethical Considerations

Experiments were carried out in accordance with the Cuban Regulations for the Use of Laboratory Animals (CENPALAB 1997) and the Canadian Council on Animal Care (CCAC) [12] This work was approved by the Ethical Committee of the International Center for Neurological Restoration. Efforts were made to minimize the pain and discomfort of the animals, as well as the number of animal used for experiments.

### 2.10. Statistical Processing

Statistical analysis was carried out using Statistical software. The values are expressed as mean ± SEM. The normal distribution and homogeneity of variance of the data were tested by the Kolmogorov–Smirnov and Levene tests, respectively. The comparisons between more than two groups were made by one-way non parametric ANOVA; the Kruskall–Wallis test was applied to aminoacid analysis, and the Wilcoxon test was applied to behavioral evaluations. In all cases, statistically significant differences were considered when *p* ≤ 0.05.

## 3. Results

Our results confirm the hypothesis that the transplantation of bone marrow mononuclear cells in the lesioned striatum of rats reverts the neurochemical alterations that appear in the neurotoxic model of Huntington’s disease induced by quinolinic acid. The objective was to evaluate the possible protective effect of transplanted mBMC on the changes induced in motor behavior and neurotransmission in the HD model.

### 3.1. Rotational Activity

**Figure 1:**
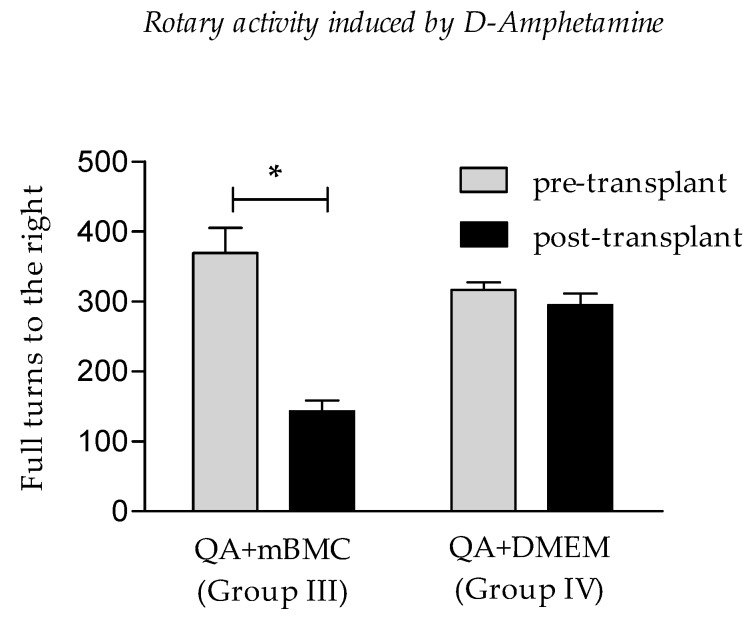
Rotatory activity induced under D-amphetamine. Lesioned group with quinolinic acid + mononuclear bone marrow cell transplant (QA+mBMC, Group III) and lesioned group with QA+Dulbecco’s modified Eagle’s medium (DMEM) transplant (QA+DMEM, Group IV). Abscissa, number of complete right turns ipsilateral to the lesioned hemisphere. Data are expressed as mean ± standard error of mean (SEM). Ordinates: experimental groups (* equivalent *p* ≤ 0.04).

Figure 1 show the ipsilateral to the lesion rotatory activity exhibited by the animals injected with QA in striatum under D-amphetamine effects. The striatal transplant of mBMC diminished the rotation induced by D-amphetamine.

### 3.2. Neurochemical Activity

#### 3.2.1. Glutamate Concentration

**Figure 2:**
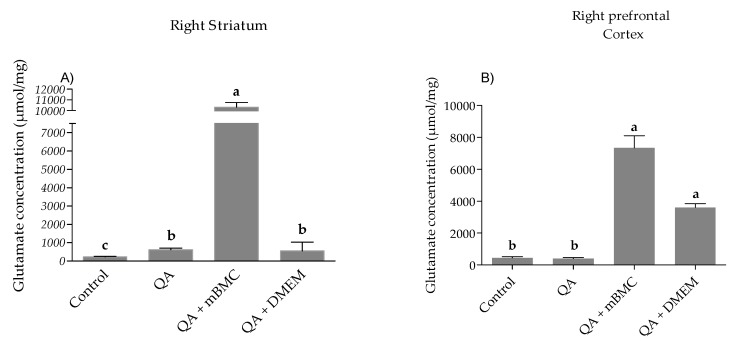
Study of glutamate (GLU) concentration in lesioned rats with transplanted mBMC. (**A**) Comparison of GLU concentration in the right striatum among the experimental groups: H (3, 20) = 16.28, *p* < 0.001. (**B**) GLU concentration in right prefrontal cortex among the experimental groups: H (3, 19) = 15.53, *p* < 0.001. Data are expressed as mean ± SEM. The letters in the top the bar correspond to statistical differences among the experimental groups. The data analysis was carried out through one-way non parametric ANOVA, using the Kruskall–Wallis test (common letters: non-significant differences; different letters; significant differences).QA lesion (Group II); lesioned group with QA + transplant (QA+mBMC, Group III); and lesioned group with QA+DMEM transplant (QA+DMEM, Group IV).

In the Figure 2 the comparison of the glutamate content in the experimental groups for the right striatum (RS) and right cortex (RC) showed significant differences among them (RS *p* ≤ 0.001 and RC *p* ≤ 0.001). The higher glutamate content was obtained for Group III (QA+mBMC) for both structures. 

#### 3.2.2. GABA (γ-Aminobutyric Acid) Concentration

**Figure 3:**
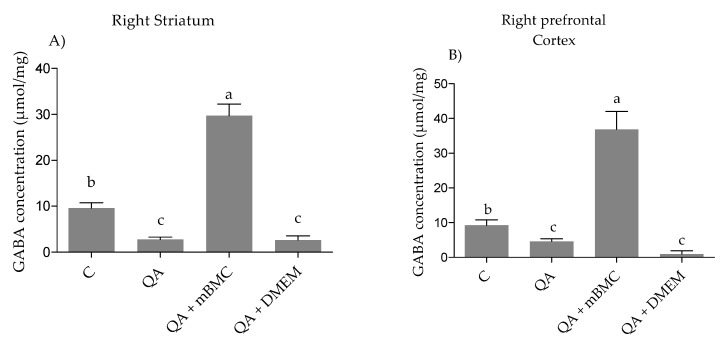
Study of GABA (γ-aminobutyric acid) concentration in lesioned rats + mBMC transplanted. (**A**) Comparison of GABA concentration in the right striatum among experimental groups: H (3, 18) = 12.55, (*p* ≤ 0.005). (**B**) GABA concentration in right prefrontal cortex among experimental group: H (3, 15) = 12.72, (*p* ≤ 0.005). Data are expressed as mean ± SEM. The letters at the top the bars correspond to statistical differences among experimental groups. The data analysis was carried out through one-way non parametric ANOVA, using the Kruskall–Wallis test (common letters: non-significant differences; different letters; significant differences). QA lesion (Group II); (lesioned group with QA + transplant (QA+mBMC, Group III); and lesioned group with QA+DMEM transplant (QA+DMEM, Group IV).

Finally, in Figure 3 the comparison of GABA (content in the right striatum (RS) and right cortex (RC) among the experimental groups showed significant differences (RS and RC: (*p* ≤ 0.001, the higher GABA content was obtained in both structures for Group III in comparison to the control group. 

## 4. Discussion

It has been suggested that striatum is the most affected structure in Huntington’s disease, with major relevance of the spiny neurons of medium size which integrate the nucleus and main receptors of the most important connections with the cerebral cortex [13,14]. The striatum is formed by different types of neural cells, but medium-sized spiny neurons, which use GABA as a neurotransmitter, represent 90–95% of the neurons found and are its main projection cells [15]. The rest of the neuronal types of the striatum are less frequent and include neurons with long dendrites that function as interneurons and use acetylcholine as neurotransmitter. The degeneration of GABAergic cells of medium size of the striatum causes the loss of influence of the neurotransmitter GABA on their targets, affecting by different pathways the “direct” and “indirect” transmission network of the motor circuit [16,17,18].

In this study, the rotational activity induced by D-amphetamine was evaluated as predictive of the degree of motor deficit present in the rats. This analysis was performed either before or after the mBMC transplant. This dose-dependent motor behavior is usually accompanied by episodes of “rotations” [19,20], which are attributed to an asymmetry of dopamine activity between the lesioned and healthy striatum [21]. The typical stereotyped behavior shown by the healthy group represents a hyperkinetic reaction characteristic of an intact brain before the action of a dopaminergic agonist (data not shown). Hence, the results of this test suggest that the damaged rats developed a rotating activity ipsilateral to the lesioned hemisphere, probably associated with the loss of GABAergic projections, as has already been described by other authors [19]. 

On the other hand, the rotational activity induced by D-amphetamine decreased significantly in the transplant group, maintaining a certain degree of rotational activity justified by the stereotypic activity caused by D-amphetamine administration. The dopaminergic imbalance observed in the present study does not respond to the decline in the dopamine synthesis process; this dopaminergic imbalance could be related to the neuronal loss of GABAergic striatal neurons which receive the nigrostriatal dopaminergic projections [21]. It is important to emphasize the fact that the rotational behavior induced by D-amphetamine in our study is only an approximate and indirect way of studying neurodegeneration in Huntington’s disease. This test only indicates the motor asymmetry that occurs in response to an imbalance in the striatum function product of the ipsilateral lesion by QA.

In this sense, previous data from our group showed that the transplant has a trophic action on the damaged tissue [22], which suggests a mechanism by which the mBMC transplant reduces the dopaminergic asymmetry between the hemispheres. The trophic support could justify the effectiveness of therapy in this area on the recovery of damaged cells, which is in line with authors agreeing on the effectiveness of mBMC transplant for improving behavior in rats lesioned with quinolinic acid [19,23].

The mechanisms by which the transplanted cells produce the recovery of normal neurochemistry in the brain are multifactorial. There is a previous report [22] with the observation that transplant cells express the NeuN protein, suggesting the transformation of them into a GABAergic neural phenotype. In another sense it is possible that the arrival of BDNF (Brain Derived Neurotrophic Factor) from other areas to the lesioned tissue contributes to the positive effect observed after the transplant. It is known that the lesion striatum can stimulate neurogenesis in the subventricular zone and produce trophic factors, including BDNF [24]. However, the possibility that the release of BDNF detected in the study reported by us [22] and that, among other actions, produces an increase in GAD (Glutamate decarboxylase), comes from the cells that are grafted and linked to the microenvironment is not excluded. On the other hand, the transplant offers an appropriate scenario to produce a positive effect in the lesioned area through the activation of other factors such as interleukins and/or growth factors. These agents guarantee the survival of nerve cells, and raise the threshold of resistance to quinolinic acid. Also, these cells are part of the immune system; protective autoimmunity effects are described as a physiological response that occurs after damage [25] that depends directly on the microenvironment where they were implanted.

Although reports of the activity of GABA and glutamate are described in experimental models [26], there is no information available on the changes in the concentration of these amino acids in the experimental model of Huntington’s disease induced by quinolinic acid and the effectiveness of mBMC transplantation.

Glutamatergic synapses are the most abundant neurotransmission systems in the central nervous system of mammals [27]. The functional complexity of these synapses is reflected at the presynaptic level by the existence of multiple excitotoxicity mechanisms initiated by the activation of autoreceptors which respond well to glutamate itself or to heterorreceptors that enhance or inhibit the release of the neurotransmitter.

The striatum begins to degenerate before other brain areas, and altered activity at corticostriatal synapses contributes to an imbalance in survival versus death signaling pathways in this brain region. Striatum projection neurons of the indirect pathway are the most vulnerable, and their dysfunction contributes to motor symptoms occurring at early stages of the disease [28]. There is evidence of expression changes in striatal excitatory synaptic activity by decreasing glutamate uptake and increasing signaling at N-methyl-d-aspartate receptors (NMDAR) [29]. 

In this study, we show the tissue concentration of the neurotransmitters as indicative of synthesis and storage processes. In addition, in the case of the striatum, this study reflects a release process because this structure is the target of cortico-striatal glutamatergic innervation. With this argument, we support the hypothesis that the increase in the glutamate content observed in the transplanted striatum is the result of all the tissue content of the neurotransmitters. Hence, the increase in glutamate in striatum and cortex observed in our results may not be deleterious for these neuronal populations, since the result of the behavior after the transplant was beneficial. More studies must be carried out to clarify this hypothesis. On the other hand, it is observed that the DMEM alone causes an induction of glutamate release. This was an expected result considering that as DMEM contains high levels of amino acids, vitamins, and glucose, it would not be surprising for some benefit to be reported after the local application of it in the lesioned tissue. For example, and in accordance with this result, beneficial effects are reported in the literature with the injection of DMEM on the maintenance and differentiation of epithelial cells [30]. However, none of these effects is as potent as that demonstrated by the mBMC transplant.

The decrease in the concentration of GABA and glutamate in lesioned animals might be related with the injury quinolinic acid and/other eventual loss of GABAergic cells [31,32,33]. Since several studies show that the administration of the neurotoxin induces cytotoxic effects on various types of neurons, which could be possibly occur though glutamate and GABA recapture systems not being intact or by damage to the blood–brain barrier. In addition, the cell transplantation could produce dendritic growth associated with the appearance of intrinsic GABAergic neuronal circuits. If we take into account the abovementioned information, it is possible that transplantation is an important factor in the establishment of the architecture and the interneuronal communication. Consequently, it modifies the physiology of the synapses of GABAergic type influencing the neurotrasmition system. 

GABAergic interneurons play a prominent role in the function of the cerebral cortex since they allow the synchronization of pyramidal neurons and greatly influence their differentiation and maturation during development [34]. A certain retrograde transport of quinolinic acid striatum–cortex with deleterious effects on the interneurons of that region may justify the decrease in GABA found in our study, although future studies must be conducted to corroborate this argument.

In this regard, changes in amino acids are likely to be the product initially of loss of GABAergic cells induced by the lesion at first stage. Previous studies conducted by our group show that transplanted cells survive for a prolonged period of time, producing a trophic action at the implant site [35]. It is also likely that this occurs after the transplant as result of the trophic action of the transplanted cells. In addition, in previous studies, we demonstrated the histopathological changes that occur in the brain lesioned with quinolinic acid and transplanted with mBMCs, through morphological and immunohistochemical studies [36,37]. The observation using cresyl violet allowed a general inspection of the striatal morphology in the different experimental groups, showing the normal cell distribution in this region. QA causes neuronal death and structural alterations, drastically reducing cell density and disrupting the pattern of cell distribution. On the other hand, our group demonstrated through an immunofluorescence study with GFAP and FJC (Fluoro-Jade C) staining that there was intense reactivity for GFAP [31,35] in the striatum of lesioned and DMEM-treated animals, indicating astrocytic gliosis in the tissue. Such a reaction was absent in the striatum of control and transplanted animals. A similar result was seen after marking with FJC, which stains degenerating neurons. Lesioned and DMEM-treated animals showed evidence of intense degeneration, which was not seen in control and transplanted animals. Finally, they were studied through immunofluorescence study by NeuN and FJC staining [36,37] whereby NeuN, a neuronal marker, showed positive reactivity in the control and the transplanted groups. In contrast, these groups were negative for FJC. The merged panel shows positive cellular bodies for NeuN in control and mBMC-transplanted groups, suggesting an integration of transplanted cells and even raising the possibility of neuronal differentiation among the transplanted cells, an aspect that we should consider in future experiments.

## 5. Conclusions

In the present study, we demonstrated that the loss of GABAergic neurons in the rat striatum as a consequence of AQ injection produces neurochemical and behavioral alterations that can be reversed with a neurorestorative treatment based on the transplantation of mononuclear bone marrow cells. Further studies will be required to understand the mechanisms by which the transplanted cells correct behavioral and neurochemical alterations produced in this model, a novel contribution of this paper.
